# Gestational Geophagia Affects Nephrocardiac Integrity, ATP-Driven Proton Pumps, the Renin–Angiotensin–Aldosterone System, and F2-Isoprostane Status

**DOI:** 10.3390/medsci7020013

**Published:** 2019-01-22

**Authors:** Emmanuel Nnabugwu Agomuo, Peter Uchenna Amadi, Chiamaka Adumekwe

**Affiliations:** 1 Department of Biochemistry, Imo State University, Owerri, Imo State 234, Nigeria; Nnabugwuago@gmail.com (E.N.A.); Chiamaka.adumekwe@gmail.com (C.A.); 2 Department of Biochemistry, University of Port Harcourt, Choba, Rivers State 234, Nigeria

**Keywords:** gestation, clay beverage, ATPase, renin–angiotensin–aldosterone, renal hemodynamics

## Abstract

Pregnancy brings about strong cravings for nonfood materials, the gestational toxicities of which are not yet ascertained. In this study, we used rat models to investigate the effect of clay beverage consumption during early and late gestation on p-Type ATPases, nephrocardiac integrity, the antioxidant system, and on the activities of the renin–angiotensin–aldosterone system (RAAS). The rats at early (7th day) and late gestation (20th) were administered single doses (500 mg/kg body weight) of clay beverage and examined using ELISA and spectrophotometry. The gestational clay beverage intake significantly elevated the renal hemodynamics, glomerular filtration rate (GFR), anion gap, urinary output, and blood urea nitrogen–creatinine ratio (BUN/Crt). At early and late gestation, clay beverage consumption elevated the heartbeat, atherogenic index of plasma, cardiac risk ratio, and atherogenic coefficients. Creatinine kinase and troponin levels after clay beverage consumption significantly increased with gestation age, while lactate dehydrogenase elevation was independent of gestation age. Mg^2+^-ATPase and Na^+^/K^+^-ATPase significantly decreased during gestation and were further altered with clay beverage intake. The rats showed higher RAAS activities during early and late gestation stages but greatly decreased activities after clay beverage administration. When F2-isoprostane and malondialdehyde levels were measured, slight elevations were found during pregnancy and were greatly elevated with clay beverage intake, while the glutathione reductase, catalase, and superoxide dismutase levels were decreased. We thus discourage clay beverage consumption throughout the entire pregnancy period because of these profound homeostatic imbalances and organ toxicities associated with its consumption.

## 1. Introduction

Pregnancy is a vulnerable and delicate period that features several physiological and anatomical changes. These changes involve almost every organ in the body and commence immediately after conception to harbor fetal development [1].

Among the greatly affected organs integral to homeostasis are the kidneys and heart. Renal physiology and hemodynamics are essentially altered during pregnancy through interstitial space expansion [2] and fluid retention [3]. A summary of the anatomical changes during gestation includes the dilation of the ureters, calyces, and pelvis, which is the primary cause of incidences of hydronephrosis that occurs in almost 90% of uncomplicated pregnancies [4]. Consequent to renal vasodilation, changes that occur in the renal physiology affect renal hemodynamics, increasing the renal plasma flow, glomerular filtration rate (GFR), urea and creatinine clearance, glycosuria, anion gap, and low blood urea nitrogen [5].

Furthermore, the increment in the metabolic demands of tissues, which consequently increases blood flow, is the major reason for the cardiovascular changes in normal pregnancy. This increase in cardiac output reduces vascular resistance and increases the heart rate, arterial compliance, and stroke volume [6]. Another important factor that undergoes profound changes during pregnancy and significantly influences gestational, renal, and cardiovascular function is the renin–angiotensin–aldosterone system (RAAS). Plasma renin activity measures the plasma angiotensin forming capacity and is regarded as the major determinant of the status of the RAAS. During pregnancy, the maternal deciduae and ovaries release extra renin, thereby increasing the renin circulating levels, which consequently increase the renin and angiotensin levels [7,8].

Since the susceptibility to renal and cardiovascular complications is higher during pregnancy, mostly via polyphagia, the consumption of most substances should be regulated throughout this period. Geophagia refers to the purposeful craving and consumption of clay, soil, and chalk, a behavior that has been documented among humans and animals for thousands of years [9,10]. Clay beverage, popularly called calabash chalk or Nzu, is one of the most commonly consumed geophagic materials in sub-Saharan Africa, a high prevalence for which has been recorded during the gestational period [11]. This substance is packed as either pellets or fine granules formed from the combination of wood ash, salt, sand, and clay, and then commercially sold in retail outlets. Pregnant women cite ethnobotanical uses, such as intended amelioration of depression, early morning vomiting, nausea, and heartburn as the major predisposing factors to clay beverage consumption [12]. Some reports in the literature have associated clay beverage consumption to altered liver parenchyma and enlarged sinusoids, the lysis of erythrocytes, intestinal hemorrhage, bone demineralization, and gestational neurotoxicities [13,14,15]. Notwithstanding the paucity of information regarding the comprehensive biological effects of clay beverage, particularly during pregnancy, there are authoritative constraints with regard to dispelling or encouraging the practice of clay beverage consumption. Thus, a better understanding of the gestational effects of clay beverage intake is availed by investigating the changes that occur to the renal and cardiovascular system, RAAS, and P-Type ATPases that represent broad range indicators of homeostatic imbalance. Consequently, we used the rat model to address this long-standing uncertainty of clay beverage consumption during early and late pregnancy, and to ascertain whether its extensive consumption would lead to complications during pregnancy.

## 2. Materials and Methods

### 2.1. Mating and Housing

Approximately 16-week-old inbred adult male and nonpregnant female Wistar rats of weights between 180 and 200g were recruited for this study. At the time of mating (after determination of estrous cycle by vaginal lavage), three female rats to one male rat were housed overnight in different plastic cages with suitable bedding and provided normal rat feed pellets (UAC Nigeria Grand Cereals Vital Feeds, Jos, Plateau State, Nigeria) and water ad libitum and tested daily for copulation. A positive vaginal smear check/mating plug confirmed pregnancy and served as the first day of gestation (G0). Normally, rats maintain an average gestation period of between 21 and 23 days, and pregnancy mainly becomes physically detectable after 2 weeks via the development of the mammary gland, abdominal changes, and weight gain. The gestation stages are divided into trimesters, where 7, 14, and 21 days represent the first, second, and third trimesters. Each recruited pregnant rat was housed individually and provided unrestricted access to rat pellets and water and handled in full compliance with the Principles of Laboratory Animal Care (NIH Publication No. 85-23), approved by the Department of Biochemistry, Imo State University Ethics Committee (IMSU/BCM/ETS/20180801).

### 2.2. Animal Diet

The nonsalted clay beverage samples used for this study were purchased in large quantities from supermarkets at Ikenegbu, Owerri, Imo State, Nigeria. The clay beverage was reduced to a fine powder using a manual grinder and dissolved in distilled water [15]. Due to its partial miscibility, the mixture was compulsorily stirred prior to oral gavage using a gastric tube. Following the method of Amadi et al. [16], a predetermined LD50 with mice occurred at >4000 mg/kg body weight. A total of five groups of ten (10) rats each was used for experimentation. All groups had unrestricted access to normal feed pellets and water. The control group consisted of nonpregnant rats fed exclusively with normal pellets and water; the second group were dams sacrificed at the 7th day of gestation (G7, early stage), the third group were dams sacrificed at the 20th day of gestation (G20, late stage), the fourth group were dams orally gavaged with 500 mg/kg b.w clay beverage and sacrificed after G7, and the fifth group were dams orally gavaged daily with1 mL of 500 mg/kg b.w clay beverage and sacrificed after G20. Body weights were measured prior to sacrifice, while urine output and heartbeat rate were measured daily. The 7th and 20th days of gestation representing early and late gestation stages were chosen as experimental durations to show if any observed biochemical effects normalize or exacerbate as pregnancy progresses. Blood samples (1 mL) were collected using a disposable syringe via cardiac puncture after pentobarbital anesthesia and serum was separated for the estimation of creatinine, electrolytes, and cardiac function markers. During separation, the collected blood was allowed to clot, and centrifuged afterwards at 4000 rpm for 10 min. The serum aliquots were transferred into a tube and freeze-stored at 20°C pending analysis. During autopsy, the heart, kidney, liver, and pancreas were dissected and weighed, while the hearts were stored in 10% formalin at −50°C for histology examination.

#### 2.2.1. Analysis of Renal Hemodynamics

Diuresis was measured as urinary output per day, while the GFR estimation was obtained from the rate of creatinine clearance. Blood urea nitrogen and creatinine were determined by colorimetry. Anion gap was calculated as the difference between the sum of serum cations (Na^+^ and K^+^) and anions (Cl^−^ and HCO_3_^−^). An Audicom (Jiangsu, China) automated electrolyte analyzer was used to determine these serum electrolytes using calculation of anion gap.

#### 2.2.2. Determination of Cardiovascular Integrity

The atherogenic index of plasma (AIP), cardiac risk ratio (CRR), and atherogenic coefficient (AC) was determined as described previously [16] as shown below:

$$\mathrm{AIP}\;=\;\mathrm{Log}\frac{Triglyceride}{HDL-C}$$

$$\mathrm{CRR}\;=\;\frac{Total\;cholesterol\;}{HDL-C}$$

AC = CRR − 1

The analysis of creatinine kinase was performed as described by Horder et al. [17], using a spectrophotometry at 450nm wavelength at 37 °C. For lactate dehydrogenase (LDH) determination, the instructions on the manual of the Lactate Dehydrogenase Kit (Sigma Aldrich St. Louis, MO, USA, Catalog No-MAK066) was carried out by enzymatic colorimetry, while the cardiac troponin levels were determined using an East Biopharm, Hangzhou, Zhejiang, China ELISA kit.

#### 2.2.3. Analysis of Total and P-type ATPases (Proton Pumps)

All reagents used were of analytical grade. Homogenates from the dissected heart, kidney, and liver of the dams, enriched in Mg^2+^-ATPase, Na^+^/K^+^-ATPase, and Ca^2+^-ATPase, were obtained as earlier described by Schulpis et al. [18]. A 10% (*w*/*v*) homogenate from the individual organs was obtained with 0.25 M ice cold sucrose using 3 × 30s Teflon Potter motor-driven homogenizer (DWK Life Sciences, Wheaton, IL, USA). The homogenate was constantly stirred and centrifuged thrice for 10 min at 10^4^g. The pellets were disposed, while the enzyme rich supernatant was used for further analysis.

The method of Schulpis et al. [18] was completely adopted for the determination of the ATP-driven-proton pumps. This method employed the measurement of inorganic phosphate released during enzymatic degradation of ATP. Briefly, the determination of total ATPase levels of the proton pump rich homogenates (100 µg) described above was carried out by introduction into a medium (1 mL) containing MgCl_2_ (4 mmol/L), Tris-HCL (50 mmol/L), KCl (20 mmol/L), anticoagulant potassium ethylenediaminetetra-acetic acid (1 mmol/L), NaCl (120 mmol/L), ATP Disodium salt (3 mmol/L) at 37°C and pH of 7.4. For the determination of Mg^2+^-ATPase, the above medium excluded NaCl and KCl but contained 1 mmol/L oubain, while Na^+^/K^+^-ATPase was obtained from the release of Pi from ATP as described by Mentzer [19]. For determination of specific Ca^2+^-ATPase activity, the result obtained using the incubation medium containing 100 mmol/L KCl, 4 mmol/L of MgCl_2_, 0.3 mmol/L ethylene glycol-bis(β-aminoethyl ether)-N,N,N′,N′-tetraacetic acid, 5 mmol/L ATP Disodium salt, and 50 mmol/L HEPES-Tris buffers was subtracted from that of a similar medium containing an addition of free Ca^2+^ (10 µM). In all proton pump determination, the reaction was stopped by adding 2 mL of 1% (NH_4_)_2_MoO_4_ in 0.9 mol/L H_2_SO_4_ after incubating for 30 min. The color developed usually partitions between yellow and orange and is read by spectrophotometry at wavelength of 390 nm.

#### 2.2.4. Measurement of the Renin–Angiotensin–Aldosterone System Activities

The plasma nenin (PRA), angiotensin (ANG), and aldosterone (ALD) activities were determined by enzyme-linked immunosorbent assay (ELISA) following the instruction protocol as described by Su-Hong et al. [20]. Serum from the experimental animals was dissolved in 0.1% of bovine serum albumin in 0.05 M Tris-buffered saline and further incubated at 37 °C for 30 mins in PRA, Ang II, ALD antibodies coated 96-well plate and afterwards washed thrice using a PBS (phosphate buffered saline) with Tween 20. Incubation further commenced at 37 °C for 30 min in a horse radish peroxidase conjugate of PRA, ANG, and ALD antibodies and afterwards washed thrice, before adding a solution of tetramethylbenzidine and hydrogen peroxide, and read at 405 nm. From the standard curve obtained for known concentrations of these proteins, the concentration of the PRA, ANG, and ALD were determined.

#### 2.2.5. Oxidative Stress Markers

Analysis of plasma 8-isoprostane was obtained by ELISA using mouse 8-isoprostane kits from Cayman Chemicals, Ann Arbor, MI, USA. The protocol descriptions were adhered to and the samples were assayed in an ELISA reader at 405 nm. Estimation of malondialdehyde (MDA) was by TBARS (thiobarbituric acid reactive substance) assay, while the glutathione reductase (GRase) levels were estimated from the decrease in absorption of NADPH [21] at a wavelength of 340 nm. The superoxide dismutase (SOD) was determined using the pyrogallol autoxidation method [22], while, as described by Aebi [23], the rate at which hydrogen peroxide decomposes within 5 min interval measured by spectrophotometry at 240nm was used as the catalase activities.

### 2.3. Statistical Analysis

All analyses were carried out in triplicates. The data obtained were analyzed using one way analysis of variance (ANOVA) with Statistical Package for Science and Social Sciences (SPSS) version 20. The statistical comparison was done among all the experimental groups for each analyzed parameter and was deemed significant at 95% confidence interval using the least standard deviations.

## 3. Results

### 3.1. Effect of Clay Beverage on Body and Organ Weights and Renal Hemodynamics during Early and Late Gestation

The body weight, organ weights, and renal hemodynamics were measured in rats administered with clay beverage during early and late gestation (Table 1). The body weights and respective organ weights of livers and kidneys significantly decreased on consumption of the clay beverage when compared with dams not administered with the clay beverage. This observed weight loss was high particularly at late gestation. In comparison to nonpregnant rats and dams not administered with clay beverage, the heart weights of dams administered with clay beverage significantly increased, while no effect was observed for the pancreas. During the gestation stages, clay beverage intake significantly and extremely decreased the urine output measured as diuresis but mildly decreased the glomerular filtration rate (GFR), followed by an extremely elevated anion gap, urea and creatinine levels.

### 3.2. Implication of Gestational Clay Beverage Intake on Cardiovascular Integrity

Table 2 shows the heart beat rate (HBR), atherogenic index of plasma (AIP), cardiac risk ratio (CRR), atherogenic coefficient (AC), creatinine kinase, troponin, and lactate dehydrogenase levels in dams administered with clay beverage. From the results, the heartbeat of the dams remained normal during the early gestation stage but significantly increased during late gestation. Dams administered with clay beverage recorded mildly but significantly higher heartbeat maintained both at early and late pregnancy with higher AIP, AC, and CRR. Additionally, gestational clay beverage intake significantly elevated the creatinine kinase and troponin levels and attained higher levels with the progression of pregnancy. The lactate dehydrogenase levels were unaltered during gestation but significantly increased after clay beverage consumption. These reported changes for the creatinine kinase, LDH, and troponin levels after geophagy were deemed excessive.

### 3.3. Relationship between Gestational Clay Beverage Intake and the Expression of Various Proton Pumps from Tissue Homogenates

**Figure 1:**
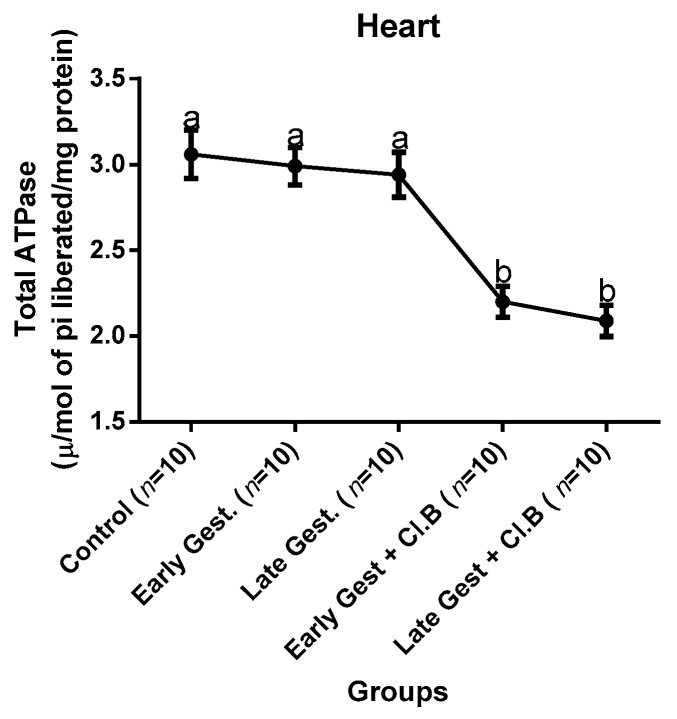
Heart total–ATPase levels after gestational clay beverage consumption.

**Figure 2:**
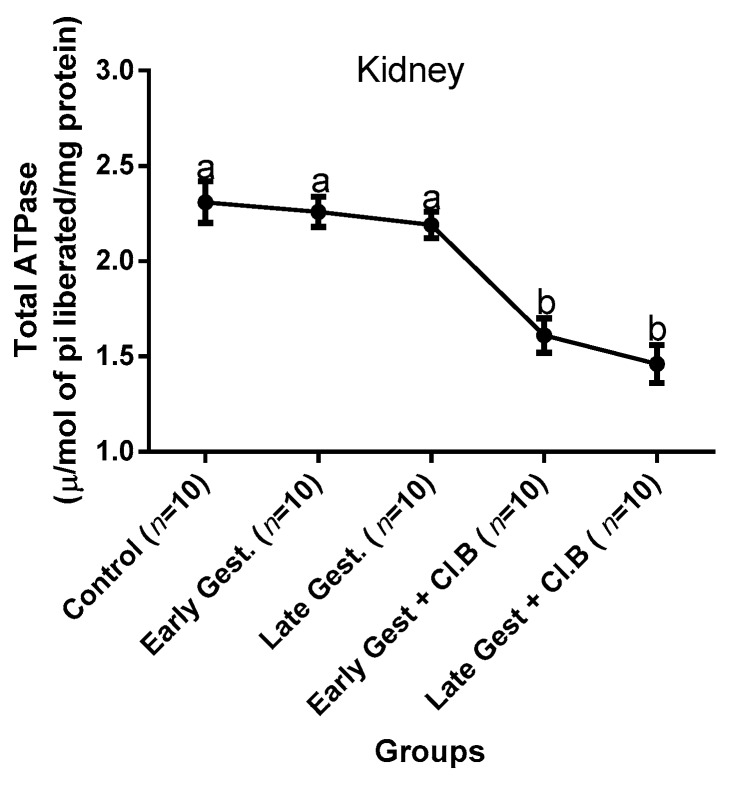
Kidney total–ATPase levels after gestational clay beverage consumption.

**Figure 3:**
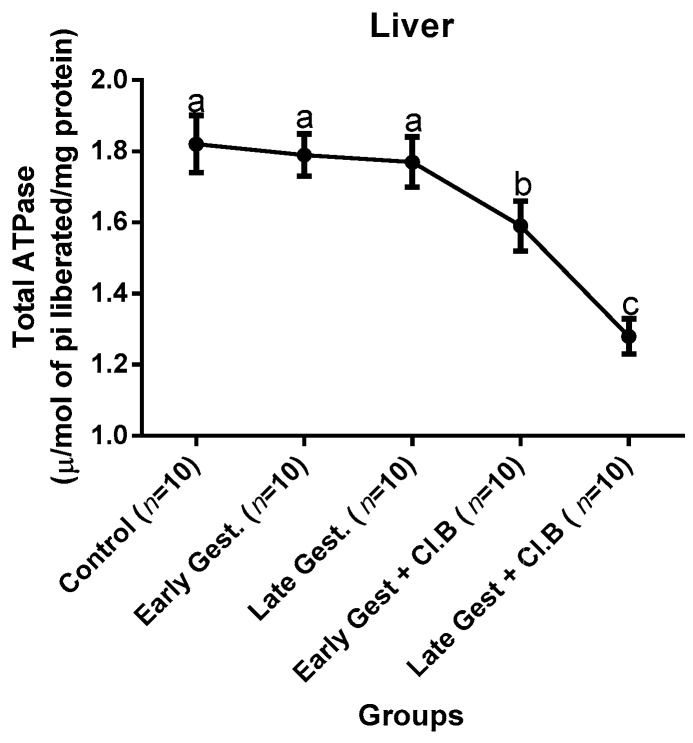
Liver total–ATPase levels after gestational clay beverage consumption.

**Figure 4:**
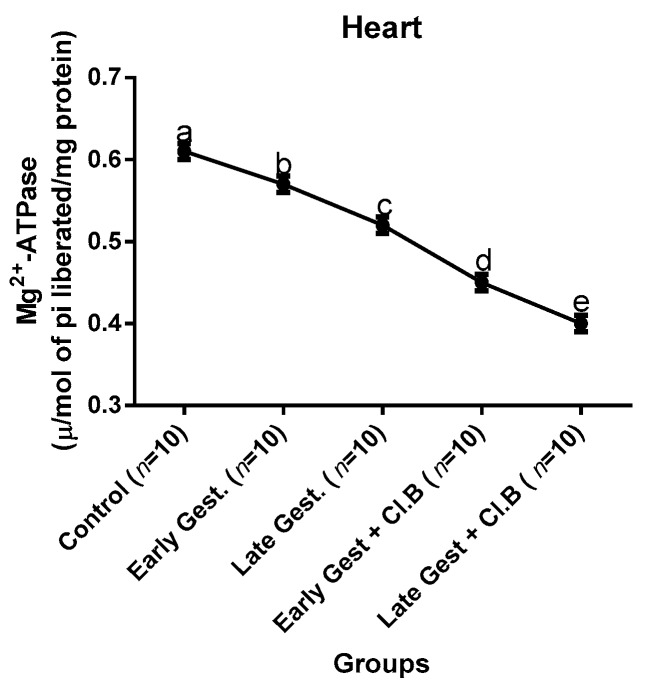
Heart Mg^2+^–ATPase levels after gestational clay beverage consumption.

**Figure 5:**
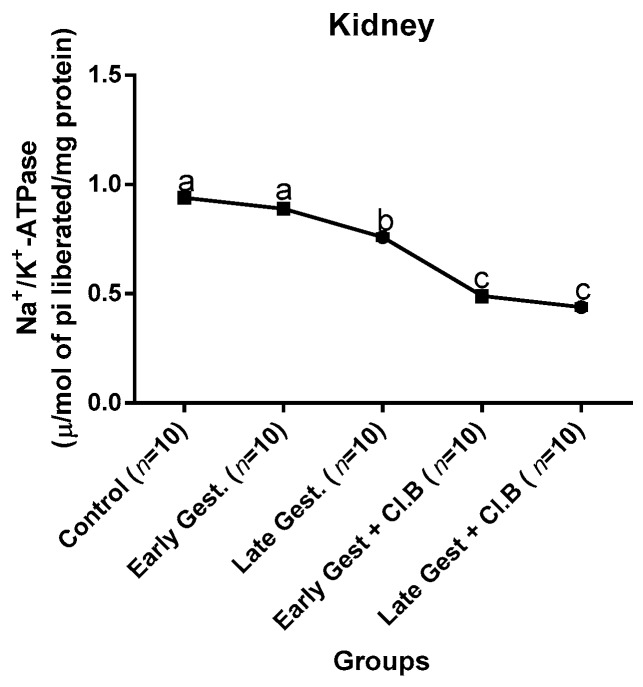
Kidney Mg^2+^–ATPase levels after gestational clay beverage consumption.

**Figure 6:**
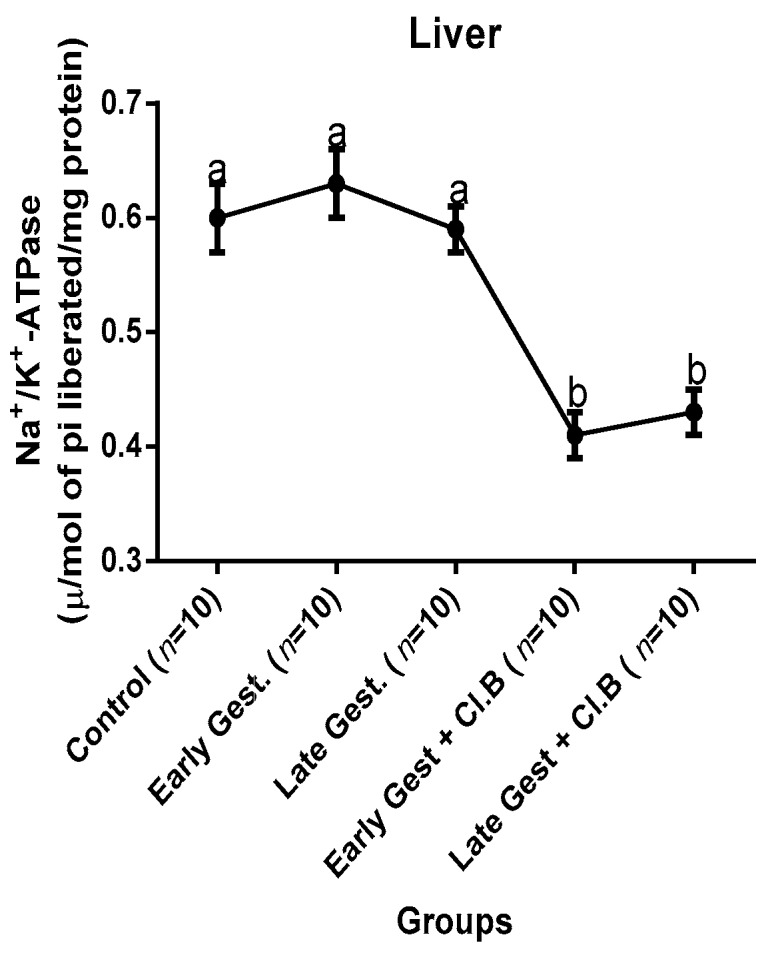
Liver Mg^2+^–ATPase levels after gestational clay beverage consumption.

**Figure 7:**
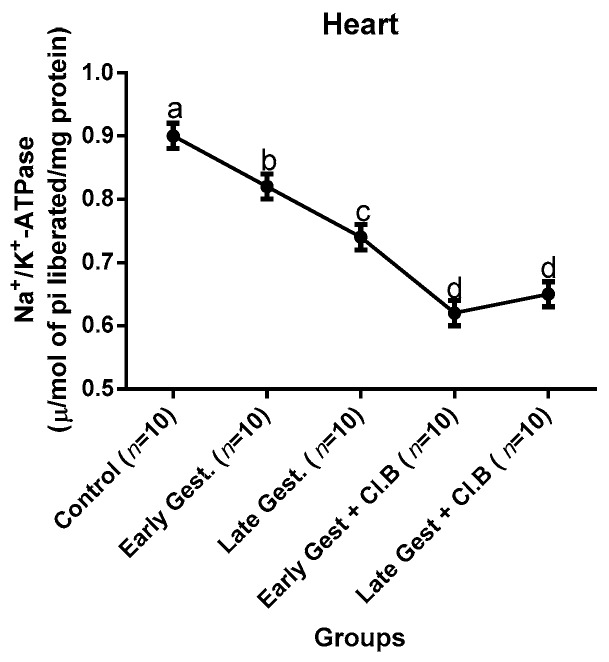
Heart Na^+^/K^+^–ATPase levels after gestational clay beverage consumption.

**Figure 8:**
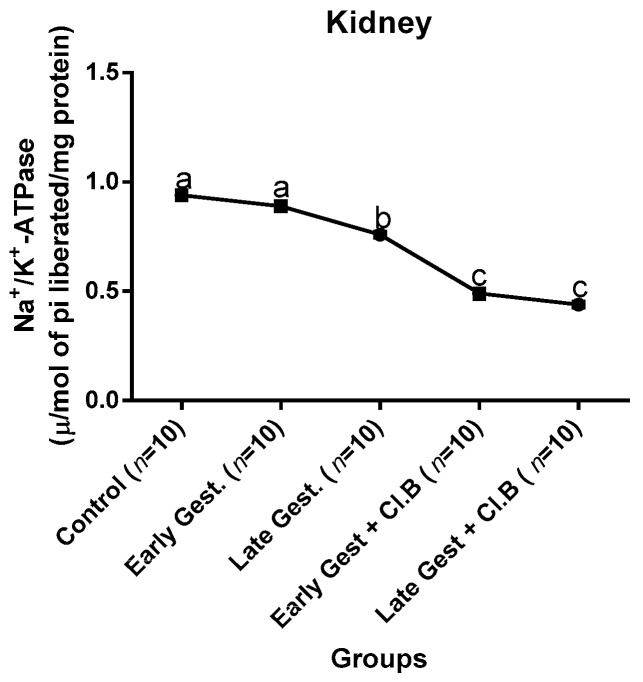
Kidney Na^+^/K^+^–ATPase levels after gestational clay beverage consumption.

**Figure 9:**
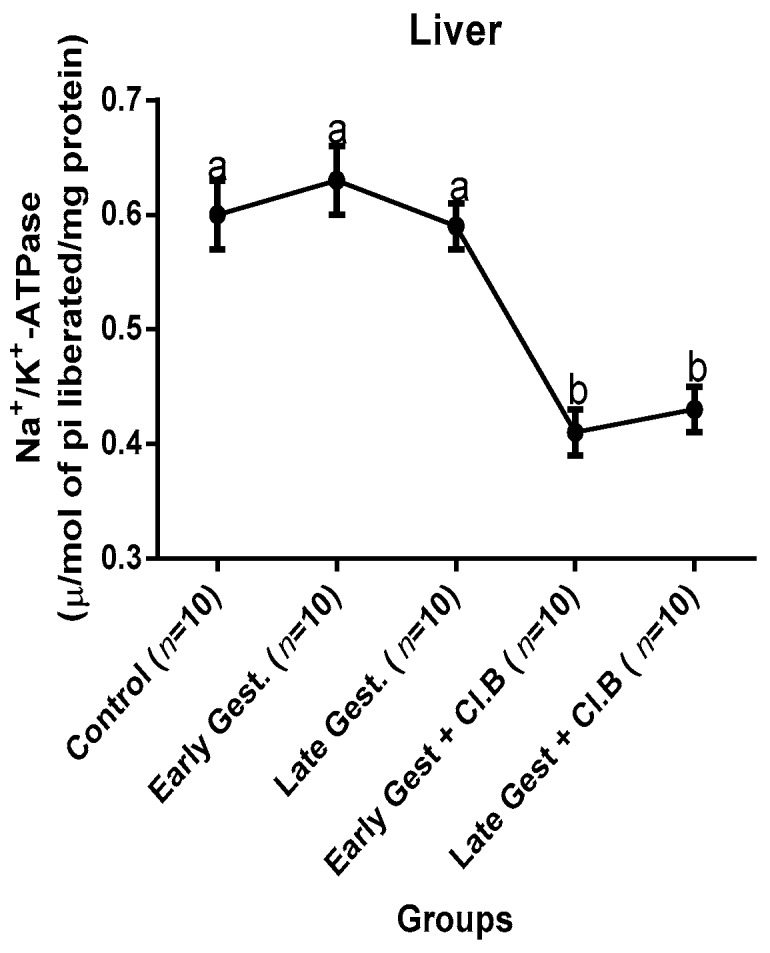
Liver Na^+^/K^+^–ATPase levels after gestational clay beverage consumption.

**Figure 10:**
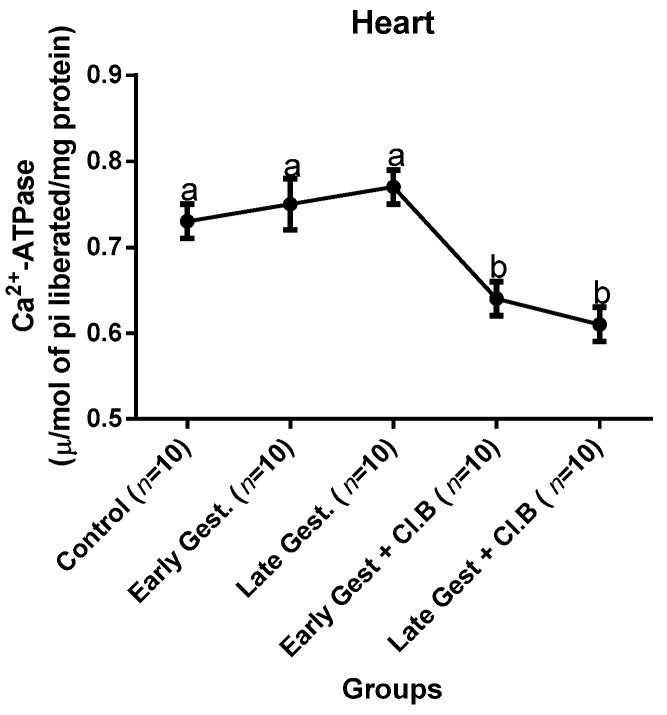
Heart Ca^2+^–ATPase levels after gestational clay beverage consumption.

**Figure 11:**
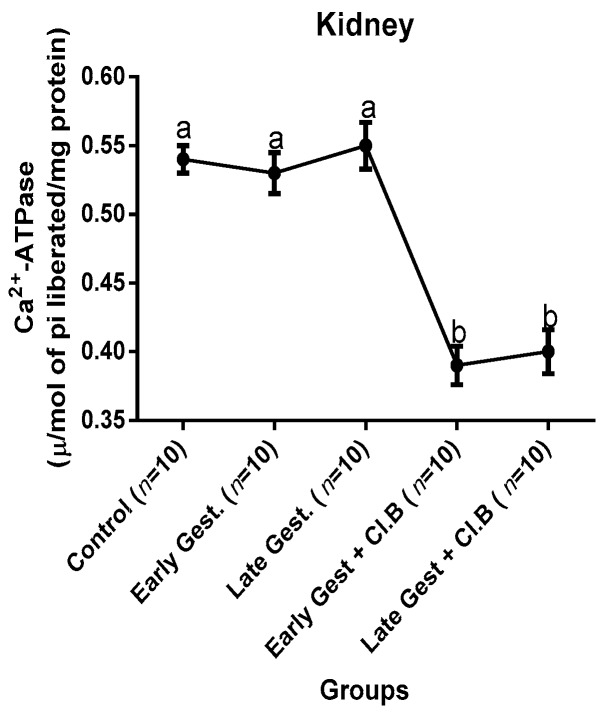
Kidney Ca^2+^–ATPase levels after gestational clay beverage consumption.

**Figure 12:**
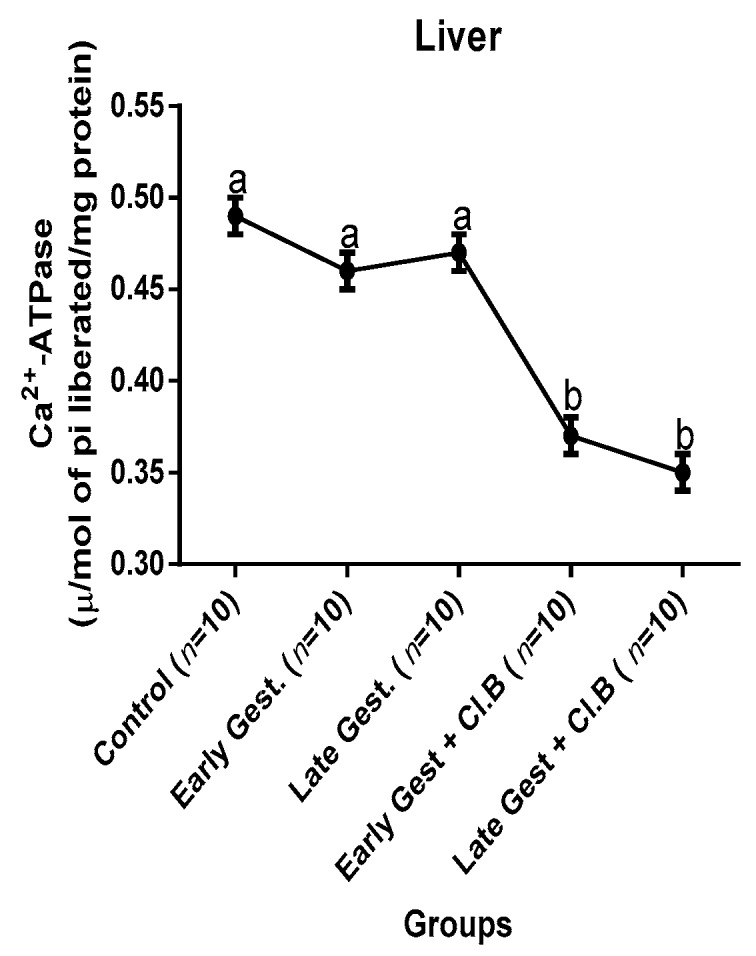
Liver Ca^2+^–ATPase levels after gestational clay beverage consumption.

The effect of clay beverage intake during early and late gestation on total ATPases, Na^+^/K^+^-ATPases, Mg^2+^-ATPases, and Ca^2+^-ATPases from heart, kidney, and liver tissues is shown in Figure 1, Figure 2, Figure 3, Figure 4, Figure 5, Figure 6, Figure 7, Figure 8, Figure 9, Figure 10, Figure 11 and Figure 12. The total ATPases from heart tissues (Figure 1) significantly decreased during gestation, but clay beverage consumption greatly suppressed the total ATPases produced by the heart, kidney (Figure 2), and liver tissues (Figure 3). The result reveals that Mg^2+^-ATPases significantly but mildly depreciates with progression of pregnancy, while clay beverage intake greatly suppressed the production of this ATPase in the heart (Figure 4), kidney (Figure 5), and liver (Figure 6). In addition, the synthesis of Na^+^/K^+^-ATPases in the heart (Figure 7) of the dams depreciated significantly during early and late gestation but significantly decreased in the kidney tissues during late gestation (Figure 8) and remained unaffected in the liver tissues (Figure 9). A continuous and excessive decline in Na^+^/K^+^-ATPases levels was noticed in dams subjected to gestational clay beverage consumption. Gestation had no observable effect on Ca^2+^-ATPases of the heart (Figure 10), kidney (Figure 11), and kidney (Figure 12) tissues but recorded a significant decline with clay beverage intake.

### 3.4. The Renin–Angiotensin–Aldosterone Activities Concentration after Clay Beverage Consumption by Rat Dams

Table 3 depicts what effect clay beverage produces on the RAAS system during early and late gestation in Wistar rats. During normal pregnancy, aldosterone was significantly elevated as the gestational stages proceeds but recorded extensive duration-dependent decline with clay beverage consumption. Other components of the RAAS system were elevated during pregnancy and remained uniform throughout gestation. Dams that consumed the clay beverage up to late gestation showed extensive decline in renin expression, whereas the clay beverage-induced reduction of the angiotensin was unaffected by gestation stage.

### 3.5. Oxidative Stress Markers and Antioxidant Enzyme Activities during Gestational Clay Beverage Consumption

In Table 4, F2-isoprostanes and malondialdehyde represent reliable oxidative stress indicators of dams administered with clay beverage. Both indicators were significantly but mildly elevated but remained comparable across the gestation stage. Clay beverage consumption by the dams slightly raised the F2-isoprostanes and malondialdehyde levels, which were also unaffected with progression to late gestation. Among the antioxidant enzymes, glutathione reductase and catalase levels of the pregnant rats were similar to those of nonpregnant rats, while superoxide dismutase levels were significantly raised during pregnancy. Clay beverage intake mildly suppressed the production of the antioxidant enzymes and remained constant throughout gestation.

**Figure 13:**
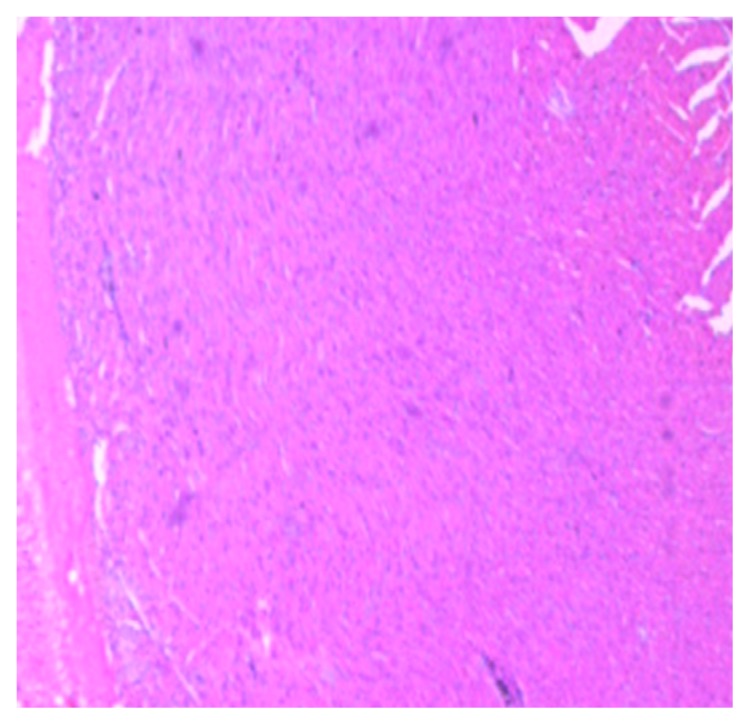
Histopathologic slide of the heart muscle of control rats showing no obvious atypical or reactive change in histology.

**Figure 14:**
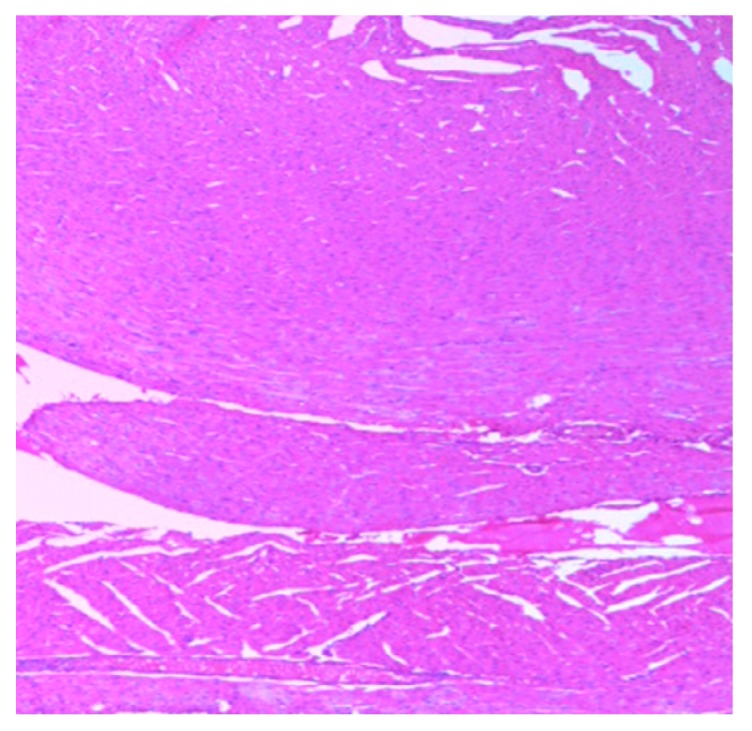
Histopathologic slide of the heart muscle of rats during early gestation stage showing no obvious atypical or reactive change in histology.

**Figure 15:**
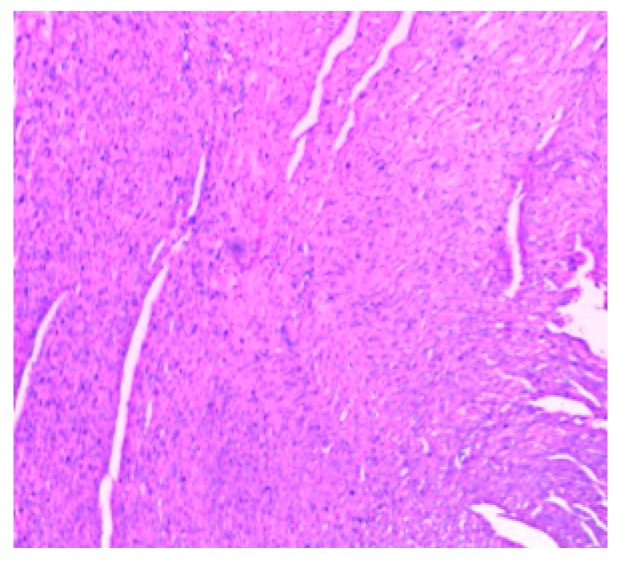
Histopathologic slide of the heart muscle of rats during late gestation stage showing no obvious atypical or reactive change in histology.

**Figure 16:**
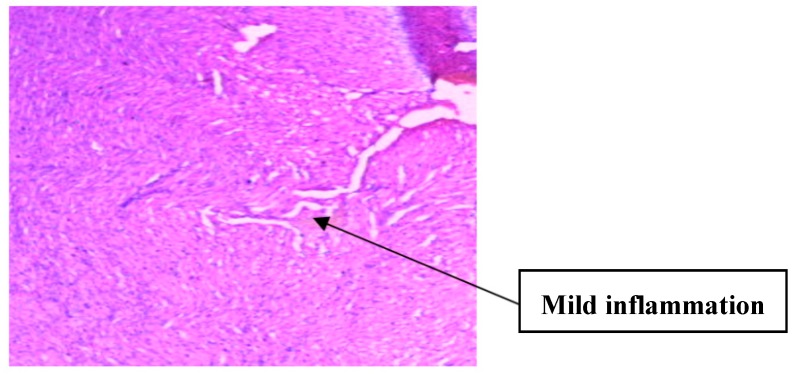
Histopathologic slide of the heart muscle of rats administered clay beverage during early gestation stage showing mild inflammation.

**Figure 17:**
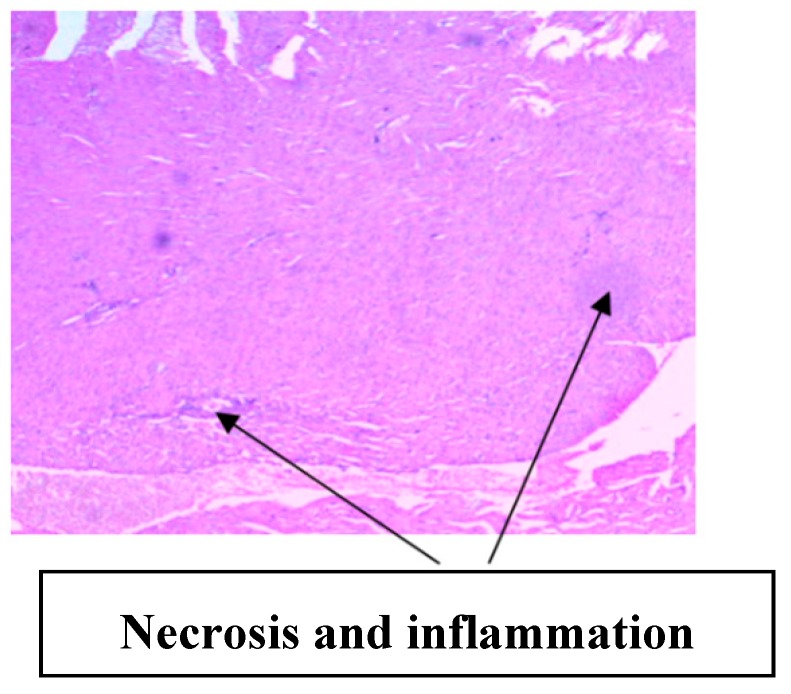
Histopathologic slide of the heart muscle of rats administered clay beverage during late gestation stage showing inflammation and necrosis.

The histology of the hearts of the rat models examined is shown in Figure 13, Figure 14, Figure 15, Figure 16 and Figure 17. Gestation had no histological changes in the cardiac tissues (Figure 14 and Figure 15), while clay beverage consumption during both early and late gestation caused inflammation (Figure 16) and necrosis (Figure 17) of cardiac tissues. We deduce that the functional integrity of the cardiovascular system is at risk during gestation if clay beverage is part of nonfood substances frequently consumed.

## 4. Discussion

Investigation of the overall homeostatic and hemodynamic effect of clay beverage, due to various health concerns associated with geophagy, became highly necessary. From our findings, by implication, the clay beverage intake during gestation led to excessive maternal weight loss following losses of major organ weights. Such sudden and excessive weight loss during pregnancy is of major concern with various clinical sequelae, indicative of toxicity and immune suppression. The weight losses could have resulted from calorie restriction, malabsorption of nutrients, or direct organ toxicities and does not bode well for both the dams and potential pups. However, it is still possible that following appropriate application, the clay beverage could be integrated as a weight loss therapy for obese pregnant women. We also observed that gestational geophagy greatly altered major parameters of renal hemodynamics. Both diuresis and the GFR measurements generally inform on the extent of proximal tubular reabsorption mechanism of the kidney, and elevated levels during pregnancy are regarded as normal renal adaptations. The altered gestational renal hemodynamics recorded in this study agree with reports elsewhere [3,24] which majorly result from pregnancy-related altered renal physiology marked with larger kidneys and consequent increase in both urine output and GFR. From suggestions of other studies [24,25], the gestational decrease in diuresis and GFR after clay beverage intake underlines renal damage and, mostly, preeclampsia. Notwithstanding that the scope of this study limited further evaluations of other inclusive criteria for preeclampsia, the suppression of renal hemodynamics, which lasted throughout the gestational stages, agrees with a detailed study conducted by Conrad and Lindheimer [26], who used the GFR as the major prognostic factor for preeclampsia. Clay beverage intake excessively evoked continuously increasing high anion gap over the gestation stages. Normal gestation physiology relates to alterations in parameters that determine pH and HCO_3_, which are electrolyte levels, fluid retention, and respiratory alkalosis, implicating the relevance of determining gestational anion gap status. Although anion gap is mostly used for examination of acid-base disorders [27], with such recorded high anion gap metabolic acidosis, gestational clay beverage consumption suggests acute nutrient deprivation [28], preeclampsia [29], hypertension [30], and acute kidney diseases [27]. In addition, the measurement of the blood urea nitrogen (BUN) and creatinine in the dams administered with clay beverage provides further backing to possible renal damage or congestive heart failure. Other studies have also shown the relevance of the blood urea nitrogen–creatinine ratio (BUN/Crt) in ascertaining renal failure, proposing that elevated BUN/Crt is diagnostic of acute renal failure [31]. Hence, this study clearly proposes that the common practice of gestational clay beverage consumption interferes with renal hemodynamics and organ integrity.

Heart rate shows the balance of parasympathetic and sympathetic activities and is a useful predictor of cardiovascular diseases [32]. During pregnancy, the high metabolic needs of the tissues account for the increased cardiac output and heartbeat to enable adequate blood flow to the organs [33]. However, abnormal elevation of resting heartbeat implies inherent cardiovascular complications [34], which may be the case for gestational clay beverage intake. Amadi et al. [16] suggest that the higher AIP, AC, and CRR, the higher the risk of cardiovascular diseases, in which case, gestational clay beverage consumption causes cardiovascular complications. AIP is indicative of plasma atherogenicity, while the AC of the dams showed that administration of clay beverage evoked higher atherogenic to atheroprotective cholesterol. Notwithstanding that some organs are regarded as creatinine kinase-rich tissues, diagnosing the etiology of elevated Crt-K is rigorous. However, according to Amir et al. [35] and Grunau et al. [36], elevated Crt-K levels in many cases indicate a damaged myocardium and, together with lactate dehydrogenase and cardiac troponin estimation, provide a sensitive diagnosis of the myocardium status. These markers, particularly troponin, are largely unaffected by normal pregnancy [37] but are elevated on account of myocardial necrosis and preeclampsia [38]. Therefore, the results strongly suggest that prolonged clay beverage intake during pregnancy predisposes the subjects to cardiovascular impairment.

The ATPases examined in our study are collectively referred to as P-Type ATPases and are crucial in the ascertainment of metabolic status and cellular processes [39] at the expense of ATP, since they primarily effect movement of protons in and out of the cell. Kherd et al. [39] proposed that these proton pumps are rarely affected during normal pregnancy, which agrees with our findings for total ATPases, while Mg^2+^ and Na^+^/K^+^- ATPases were suppressed particularly in kidneys and hearts and mostly during late gestation. The implication of the decline in the P-Types ATPase is alterations in ionic balance of the cellular matrix and, on this account of clay beverage intake, testifies to its necrotic potentials on the various organs. Thus, the decline in expression of these ATP driven proton pumps after gestational clay beverage consumption implies chronic homeostatic imbalance by primarily targeting the functional integrity of the heart, kidney, and liver.

The RAAS system is a pivot to the regulation of blood pressure, electrolyte balance, and perfusion of the placenta, whereby the gestational elevation of the RAAS is consequent to adaptation to the increment in blood flow, pressure, and volume and is thus regarded as a normal outcome during pregnancy [40]. Świątkowska-Stodulska et al. [41] reported that the elevated RAAS activities from the first trimester of normal pregnancy result from hyperestrogenemia and hyperprogesteronemia and increase up to 3 times towards the end of gestation. There are studies suggesting the sudden perturbation of the RAAS during pregnancy in cardiovascular disorders [42]. In the study of Malha et al. [43], the renin and aldosterone levels of hypertensive pregnant women were lower than those for normotensive pregnant women, which, thus, in this study, implicates the deregulation of renin by the clay beverage as a potential source of hypertension. In addition, the hypertensive tendency of clay beverage intake is evident from the extensive angiotensin suppression. Angiotensin, similar to renin, impacts on the homeostasis of blood pressure, and its suppression testifies to cardiovascular anomalies. More recently, Tutunea-Fatan et al. [44], using shGRK2 knockdown mice, confirmed that the suppression of angiotensin expression was etiogenic to the pathophysiology of cardiovascular disorders; thus, gestational clay beverage consumption has shown potentials of altering cardiovascular integrity.

The measurement of F2-isoprostanes was pertinent to understand the effect of geophagy on the antioxidant system. F2-isoprostanes are prostanglandin-like compounds generated during the peroxidation of essential fatty acids by free radicals. The slight elevation in F2-isoprostane and malondialdehyde levels as observed in our study is a normal feature during pregnancy [45,46,47]; however, excessive production of these oxidative stress markers basically indicates oxidative damage [48]. Antioxidant enzymes are only suppressed during oxidative stress-related diseases caused particularly by toxic exogenous substances [16]. By implication, the pregnant rats had a compromised defense system due to prolonged clay beverage intake up to the late gestation stage.

## 5. Conclusions

The gestational clay beverage intake has been examined across various organ functions. At both early and late gestation, clay beverage intake altered the renal hemodynamics and greatly provoked excessive urinary output and abnormal glomerular filtration rate. Its consumption was seen to negatively affect the cardiovascular system by increasing the heartbeat, cardiac triponin, creatinine kinase, and cardiac risk ratio. Further disruption of the membrane proton pump ATPases, the renin–angiotensin–aldosterone system, and oxidative stress defense mechanism confirms the potentials of gestational clay beverage consumption to induce homeostatic imbalance and nephrocardiac disorders. Thus, in light of these findings, the potentials of clay beverage to induce pre-eclampsia as well as its teratogenic effect require imminent investigation.

## Figures and Tables

**Table 1:** Morphometrics and renal hemodynamics of pregnant rats administered clay beverage.

Parameters	Control (*n* = 10)	E.G (*n* = 10)	L.G (*n* = 10)	E.G + Cl.B (*n* = 10)	L.G + Cl.B (*n* = 10)
BW (g)	195.36±7.26^a^	253.40±6.44^b^	296.05±7.23^c^	216.21±4.45^d^	237.50±5.21^e^
LW (g)	9.45±0.62^a^	11.31±0.79^b^	12.81±1.05^b^	9.41±0.40^a^	9.83±0.62^a^
KW (g)	1.94±0.12^a^	1.99±0.20^a^	1.92±0.33^a^	1.84±0.57^b^	1.79±0.44^b^
HW	1.46±0.11^a^	1.62±0.15^a^	1.50±0.13^a^	1.96±0.17^b^	1.83±0.13^b^
PW	0.93±0.08^a^	0.90±0.07^a^	0.92±0.06^a^	0.94±0.07^a^	0.95±0.11^a^
DIU (mL)	4.97±0.21^a^	7.25±0.55^b^	7.57±0.48^b^	3.08±0.33^c^	2.59±0.35^c^
GFR *	0.63±0.06^a^	0.84±0.09^b^	0.89±0.07^b^	0.77±0.08^c^	0.70±0.08^c^
Anion gap	9.97	9.76	10.07	14.18	18.50
BUN (mg/dL)	27.01±1.73^a^	20.80±2.12^b^	18.11±1.19^b^	37.36±3.94^c^	39.22±2.04^c^
Creatinine (mg/dL)	0.52±0.07^a^	0.44±0.05^b^	0.41±0.06^b^	0.66±0.09^c^	0.69±0.05^c^
BUN/Crt	51.94	47.27	44.17	56.60	56.86

* mF/min/100g bw. Values are means ± S.D of triplicates. Values with different superscript letter(s) (a–e) across the row are significantly different (*p* < 0.05). E.G—early gestation, L.G—late gestation, Cl.B—clay beverage, BW—body weight, LW—liver weight, KW—kidney weight, HW—heart weight, PW—pancreas weight, DIU—diuresis, GFR—glomerula filtration rate, BUN—blood urea nitrogen, BUN/Crt—blood urea nitrogen–creatinine ratio, *n*—number of rats.

**Table 2:** Markers of cardiovascular integrity after gestation clay beverage consumption.

Groups	HBR (Beat/Min)	AIP	CRR	AC	CRT-Kin(U/L)	TROP(ng/mL)	LDH (U/L)
Control (*n* = 10)	320.5±9.5^a^	0.25	2.73	1.73	377.1±11.7^a^	3.40±0.4^a^	248.3±14.4^a^
E.G (*n* = 10)	327.8±8.6^a^	0.30	2.90	1.90	382.1±8.9^a^	3.51±0.6^a^	239.4±17.1^a^
L.G (*n* = 10)	349.4±10.1^b^	0.31	3.00	2.00	390.8±8.6^a^	3.47±0.4^a^	259.4±11.9^a^
E.G + Cl.B (*n* = 10)	372.3±7.7^c^	0.53	4.74	3.74	443.9±14.1^b^	4.82±0.6^c^	329.7±19.3^b^
L.G + Cl.B (*n* = 10)	380.4±11.8^c^	0.46	4.51	3.51	470.2±16.6^c^	4.99±0.5^d^	302.4±16.9^b^

Values are means ± S.D of triplicates. Values with different superscript letter(s) (a–d) across the row are significantly different (*p* < 0.05). E.G—early gestation, L.G—late gestation, Cl.B—clay beverage, HBR—heart beat rate, AIP—atherogenic index of plasma, CRR—cardiac risk ratio, AC—atherogenic coefficients, CRT-Kin—creatinine kinase, Trop—cardiac troponin, LDH—lactate dehydrogenase, *n*—number of rats.

**Table 3:** Effect of clay beverage consumption during early and late gestation on rennin–angiotensin–aldosterone system.

Groups	REN (ng/mL)	ALD (ng/mL)	ANG (pg/mL)
Control (*n* = 10)	61.8±5.5^a^	177.2±13.5^a^	76.6±5.1^a^
E.G (*n* = 10)	97.4±6.1^b^	286.6±18.4^b^	104.9±7.2^b^
L.G (*n* = 10)	92.2±8.5^b^	317.2±16.0^c^	99.4±8.0^b^
E.G + Cl.B (*n* = 10)	50.5±5.9^c^	94.1±7.7^d^	53.6±4.2^c^
L.G + Cl.B (*n* = 10)	43.3±4.7^d^	82.5±8.2^e^	55.8±3.9^c^

Values are means ± S.D of triplicates. Values with different superscript letter(s) (a–e) across the row are significantly different (*p* < 0.05). E.G—early gestation, L.G—late gestation, Cl.B—clay beverage, REN—renin, ALD—aldosterone, ANG—angiotensin, *n*—number of rats.

**Table 4:** Effect of clay beverage consumption during early and late gestation on oxidative stress indicators.

Group	F2-Isoprostane (pg/mL)	MDA *	GRase **	CAT (U/mg)	SOD (U/mg)
Control (*n* = 10)	21.4±0.04^a^	0.48±0.07^a^	7.50±1.7^a^	59.17±5.3^a^	15.50±2.1^a^
E.G (*n* = 10)	26.9±0.04^b^	0.59±0.06^c^	7.71±1.1^a^	61.91±3.9^a^	19.26±1.8^b^
L.G (*n* = 10)	28.0±0.03^b^	0.62±0.07^c^	8.04±1.5^a^	60.27±3.1^a^	21.49±2.4^b^
E.G + Cl.B (*n* = 10)	37.3±0.06^c^	0.79±0.08^d^	6.13±1.3^b^	46.52±5.9^b^	8.30±1.4^c^
L.G + Cl.B (*n* = 10)	35.4±0.05^c^	0.83±0.06^d^	5.93±0.9^b^	43.80±4.4^b^	8.09±0.8^c^

* = nmol/mg protein, ** = nmol NAPDH consumed/min/mg protein. Values are means ± S.D of triplicates. Values with different superscript letter(s) across the row are significantly different (*p* < 0.05), *n*—number of rats.
